# Direct Evidence of Bimolecular Proton-Coupled Energy Transfer at Room Temperature

**DOI:** 10.1021/jacs.5c05126

**Published:** 2025-07-03

**Authors:** Andrea Rosichini, Giorgio Scattolini, Leif Hammarström

**Affiliations:** Department of Chemistry, Ångström Laboratory, 8097 Uppsala University, Box 523, 75120 Uppsala, Sweden

## Abstract

A new elementary reaction, denoted proton-coupled energy transfer (PCEnT), has been recently reported in a series of donor–acceptor molecules. In this reaction, excited state energy transfer is made possible by a simultaneous transfer of a proton on the energy acceptor. This type of elementary reaction could have, by analogy to proton-coupled electron transfer, an important role in photochemistry and energy transportation of biological systems. In the previously reported case, the reaction is shown to occur intramolecularly in a covalently linked system in a 77 K glass. In this work, we identify a suitable bimolecular system for PCEnT and provide direct evidence for PCEnT in a room temperature solution using fluorescence spectroscopy. Based on these results, we discuss some simple design principles for PCEnT, including some of the current obstacles in designing a successful system.

## Graphical abstract

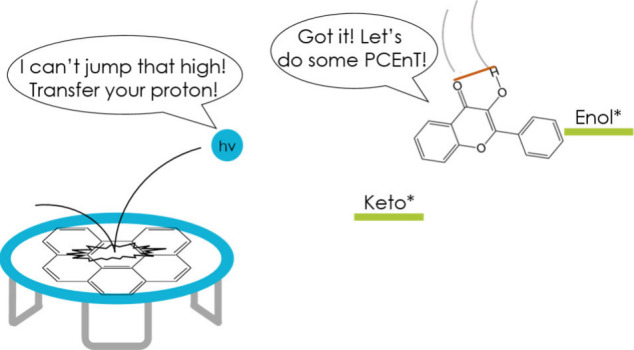

Electronic excitation energy transfer (henceforth: “energy transfer”) is a fundamental elementary reaction in which the energy of an electronic excitation is transferred from a donor to an acceptor. This reaction is common in nature, in particular in photosynthetic organisms, where the energy of absorbed photons is transported via energy transfer between pigments.^1,2^

An elementary reaction where energy transfer is coupled to proton transfer in one concerted step was recently discovered in covalently linked donor–acceptor (D–A) molecules and denoted Proton-Coupled Energy Transfer (PCEnT).^3^ Light excitation of an anthracene unit led to PCEnT, forming an excited state of the phenol-pyridine (2-(pyridine-2-yl)­phenol) acceptor unit, which was linked to anthracene via a methylene bridge. The state formed was the excited keto-tautomer, detected by its fluorescence in a rigid matrix, in which the phenolic proton had transferred to the pyridine. The same state can be formed by direct excitation of phenol-pyridine, followed by a tautomerization in the excited state known as Excited State Intramolecular Proton Transfer (ESIPT).^4^ Direct excitation, however, would require at least 0.5 eV more energy than what was available in the excited anthracene. Similarly, proton transfer prior to energy transfer would have been >0.5 eV uphill, showing the need for a concerted transfer of proton and energy. The role of proton transfer in lowering the energetics of the final product, and opening reaction pathways that would otherwise not be possible, has been well documented for Proton-Coupled Electron Transfer (PCET),^5,6^ and the case is analogous for PCEnT.^7^ Given the conceptual similarities between PCEnT and PCET, an isomorphic theoretical framework for PCEnT has been recently proposed.^7^ In the D–A molecules, there was no observable spectral overlap between donor emission and acceptor absorption, as required for Förster energy transfer, and the donor–acceptor distance was short. Therefore, a Dexter mechanism (or other short-range coupling terms) was proposed.^3^

In this work we aimed to identify new systems capable of PCEnT, to understand if this type of reaction requires the very specific conditions present in the original D–A molecules, or if it is a more generally occurring reaction. Furthermore, we aimed to gain more mechanistic insight into the PCEnT reactions and the conditions that make them possible.

Differently than in the original study on D–A molecules, all the acceptors used exhibit room temperature fluorescence in fluid solution,^8–10^ which allows PCEnT to be studied in bimolecular reactions. This introduced some differences compared with the previously studied linked systems: (1) Multiple combinations of donors and acceptors can be explored to test if PCEnT can be a generally occurring phenomenon, without the need for a new synthesis for each pair. (2) The system is not covalently linked; therefore the distance between donor and acceptor is not fixed. This allows for probing if a covalent link between the donor and acceptor is a requirement for PCEnT. Specifically, the short methylene link of the previous D–A molecules may have promoted through-bond or through-space electronic coupling between donor and acceptor. It may also have promoted vibronic coupling between the units, e.g., collective vibrational modes (see ref 7). While they could still be present in the bimolecular encounter complexes of the present study, their nature would be different compared to those of the covalently linked D–A molecules. (3) The maximum possible rate constant is, however, limited by diffusion of the reactants.

The systems studied were selected using design principles mostly based on energetic arguments. The energy levels of the donor and acceptor involved in a PCEnT reaction can be schematically visualized through the energy diagram in Figure 1.

**Figure 1:**
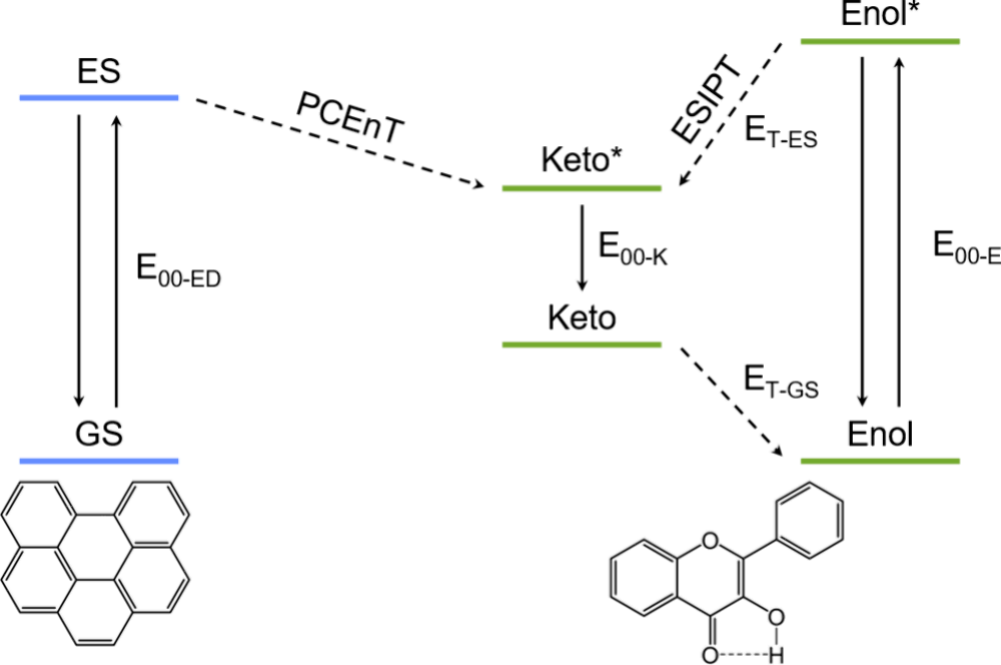
Energy levels of the donor (blue) and acceptor (green) molecules involved in a PCEnT reaction.

For PCEnT to be energetically feasible, the excited state energy of the donor molecule (*E*_00‑ED_) needs to be higher than the excited state energy of the keto form of the acceptor (*E*_00‑K_) plus the ground state tautomerization energy (*E*_T‑GS_), i.e., the keto*–enol energy difference. Obtaining a value for this energy is probably the most challenging step in designing a PCEnT system. In fact, while the excited state energies of both the enol and keto tautomer (*E*_00‑E_ and *E*_00‑K_) can be experimentally estimated from the enol absorption and keto fluorescence of the molecule, respectively, *E*_T‑GS_ is hard to obtain experimentally, and the values reported in the literature are obtained from computations. Due to specific solvent interactions of the ESIPT molecules, the computed tautomerization energies are often unreliable and can differ by hundreds of meV between different calculation methods.^11–14^

In addition, to avoid pure energy transfer followed by rapid ESIPT in a stepwise mechanism, which would be difficult to distinguish experimentally from PCEnT, we selected donors with excited state energies lower than the excited state energy of the enol form of the acceptor (*E*_00‑E_).

In this study we combine two different singlet energy donors (perylene and benzo­[*ghi*]­perylene) with four different ESIPT compounds as energy acceptors: 3-hydroxyflavone (3HF), 10-hydroxybenzoquinoline (HBQ), and two phenol-*n*-quinolines (P5Q, P6Q)^8^ (structures shown in Figure S1).

Due to its short singlet lifetime (4 ns), the experiments with perylene were performed confining donor and acceptor in cetyltrimethylammonium chloride (CTAC) micelles to avoid using high bulk concentrations of the quenchers (Figures S2–S6). The experiments with benzo­[*ghi*]­perylene were instead performed in solution, since its longer singlet lifetime (97 ns) allows for diffusional quenching at relatively low acceptor concentrations (<1 mM).

Among the donor–acceptor pairs studied, only the benzo­[*ghi*]­perylene–3HF pair showed evidence of PCEnT, and we will present and discuss the results for this pair in the remainder of this text. The results for the other pairs are shown in the SI, and potential reasons for the absence of PCEnT in those systems will be discussed below.

The pair benzo­[*ghi*]­perylene–3HF was studied in four different deoxygenated aprotic solvents with different polarities (decane, tetrahydrofuran (THF), and acetonitrile) and with conjugated π-bonds (toluene) and in a polar protic solvent (ethanol).

In all solvents studied, we observed quenching of the fluorescence of benzo­[*ghi*]­perylene in the presence of 3HF. Enhancement of the keto fluorescence of 3HF in the presence of the donor was also observed (Figure 2) in every solvent, except ethanol. This simple observation provides direct evidence for a PCEnT reaction. Since the ground state keto tautomer cannot be thermally populated, and stepwise energy transfer to form enol* followed by proton transfer can be excluded on energetic grounds (*E*_00‑E_ ≈ 3.3 eV;^15^ *E*_00‑ED_ = 3.05 eV^16^ from literature, ≈3.15 eV from the crossing point in Figure S7c), we can assign the reaction as concerted PCEnT.

**Figure 2:**
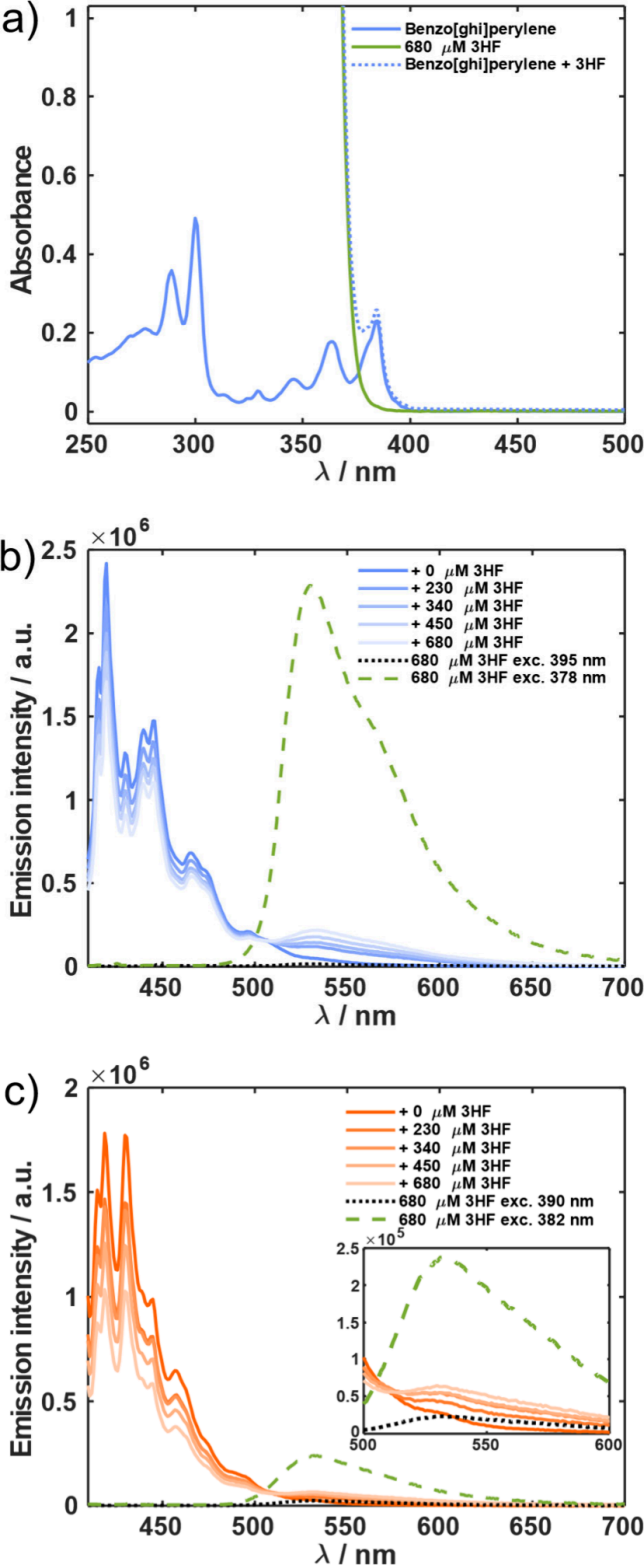
a) Absorption spectra of the benzo­[*ghi*]­perylene/3HF pair and of the individual components in decane. b) Steady state fluorescence quenching of benzo­[*ghi*]­perylene by 3HF in decane. c) Steady state fluorescence quenching of benzo­[*ghi*]­perylene by 3HF in acetonitrile. Dotted and dashed lines in panels b) and c) show the fluorescence of 3HF on its own at the excitation wavelength used for the donor (390 nm) and at an excitation wavelength where the acceptor has the same absorbance as the donor at 390 nm.

No quenching was observed in control experiments with methoxy-3HF, where the hydrogen-bonded proton is replaced with a methyl group (Figure S11). This confirms that transfer of the hydrogen-bonded proton is needed for the energy transfer to happen.

A Stern–Volmer analysis yielded near-diffusion-limited quenching rate constants for all the solvents studied (Table 1). From transient emission kinetic measurements (Figure S17), we verified the quenching to be entirely dynamic in acetonitrile, while both a static and a dynamic component seems to be present in decane (Table 1).

**Table 1:** Rate Constants for Quenching of Benzo­[*ghi*]­perylene Fluorescence by 3HF in Different Solvents, Calculated from Steady State and Transient Experiments^a^

		From steady state data		From transient data at 680 μM 3HF	
Solvent	τ_0_ (ns)	*k*_SV_ (M^–1^)	*k*_q_ (M^–1^ s^–1^)	*k*_obs_ (s^–1^)	*k*_q_ (M^–1^ s^–1^)
Decane	97	623	6.40 × 10^9^	1.23 × 10^7^	2.91 × 10^9^
Toluene	-	329	3.39 × 10^9^	-	-
THF	-	300	3.09 × 10^9^	-	-
Acetonitrile	116	1050	9.05 × 10^9^	1.53 × 10^7^	9.50 × 10^9^

a A detailed description of the calculations can be found on page S15 of the SI. Stern–Volmer plots for all solvents are shown in Figure S10.

Transient absorption (TA) spectra of benzo­[*ghi*]­perylene in decane, in the presence of 3HF, result in an additional negative feature around 550 nm not present for benzo­[*ghi*]­perylene alone. This can be assigned to the fluorescence of the keto form of 3HF (Figure S14). It disappears with the same lifetime as that of benzo­[*ghi*]­perylene fluorescence, consistent with a keto* lifetime (<10 ns^17^) much shorter than the time of its formation (*k*_obs_^–1^; Table 1). A longer-lived feature, observed both with and without 3HF, that survives for tens of μs (Figure 3 and Figure S15) is consistent with the triplet state spectra of benzo­[*ghi*]­perylene.^18^ In acetonitrile, we observe additional spectral features at 400 and 505 nm (Figure 3) with lifetimes of tens of microseconds (Figure S15). We assign the peak at 505 nm to the reduced form of benzo­[*ghi*]­perylene by comparison with the TA spectra obtained from reductive quenching of benzo­[*ghi*]­perylene with *p*-anisidine (details in the SI). This suggests that the feature at 400 nm is due to oxidized 3HF. The combination of fluorescence and TA results indicate the presence of two competitive pathways in acetonitrile, which can be assigned to PCEnT and PCET. PCET, generating charged products, would be favored by an increase in solvent polarity, whereas the more charge-neutral PCEnT can dominate in nonpolar solvents. A similar solvent-polarity dependence of PCET vs PCEnT was recently reported for the covalently linked D–A molecule.^19^ In ethanol, being both polar and protic, we observe no PCEnT (Figure S13). However, since the PCEnT reaction was close to diffusion controlled in all other solvents, it was not possible to determine how the intrinsic PCEnT rate constant depends on the solvent polarity.

**Figure 3:**
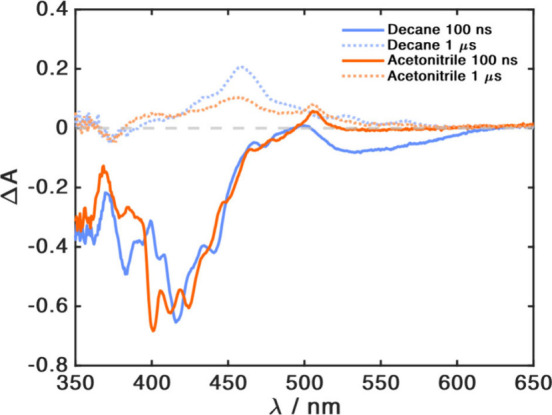
Transient absorption spectra of a solution of benzo­[*ghi*]­perylene (20 μM) and 3HF (700 μM) in decane (blue) and acetonitrile (orange), excited at 390 nm, and measured 100 ns (continuous lines) and 1 μs (dashed lines) after the excitation. In the spectra measured at 100 ns, the negative features above 390 nm originate from spontaneous emission.

These results show that PCEnT in bimolecular systems is possible, and the reaction can be intrinsically fast even when the donor and acceptor are not covalently linked or preassociated. The near-diffusion-limited rate constant and the absence of spectral overlap between donor fluorescence and acceptor absorption spectra point toward a Dexter or other short-range mechanism. However, recent theoretic work has suggested that PCEnT can occur through a Förster mechanism even in the absence of experimentally observable spectral overlap between the donor emission and the acceptor absorption.^7^

The pair benzo­[*ghi*]­perylene–3HF is the only one that successfully shows PCEnT out of the ones tested. Understanding why the other pairs did not work can provide precious insight on how to design successful PCEnT systems.

The first thing to consider, as mentioned above, is the lack of reliable values for the tautomerization energies for most ESIPT compounds. Taking the example of 3HF, *E*_T‑GS_ computed values can vary by up to 250 mV (from 0.43 to 0.68 eV).^12,13^ This complicates the evaluation of the correct energetic requirements for the energy donors.

For example, the excited state energy difference between benzo­[*ghi*]­perylene (3.05 eV)^16^ and perylene (2.84 eV)^20^ is 210 mV. Depending on the set of computed values used, PCEnT can vary from energetically downhill^12^ to energetically uphill for both energy donors.^13^

In addition, the pairs having perylene as the donor were studied in CTAC micelles. We show that both 3HF fluorescence intensity and PCEnT are dependent on solvent polarity, with intensity becoming weaker in more polar solvents (Figure S17) as the weak internal hydrogen bond of 3HF is weakened.^9,15,21^ As the fluorescence intensity of 3HF in CTAC micelles is between the intensity in acetonitrile and in ethanol, 3HF experiences a polar environment. While we observe quenching without acceptor fluorescence enhancement, that can be tentatively assigned to PCET, for some of the studied pairs in CTAC, the benzo­[*ghi*]­perylene–3HF pair exhibits no quenching, indicating that PCET is also hindered (Figure S12). This result is still puzzling.

It is worth noting that HBQ, on the other hand, has been reported to have a very strong hydrogen bond, fairly insensitive to solvent polarity and hydrogen-bonding properties.^10,22^ Therefore, the absence of PCEnT in the experiments with HBQ can be found in the energetic arguments discussed above.

These results prove that PCEnT is possible in systems different than the original D–A molecules and can happen bimolecularly. The discovery of a new elementary reaction opens interesting perspectives for research. For example, it would be interesting to understand the role of PCEnT in natural systems and the potential applications in artificial systems such as molecular photoswitches.^23,24^ For a deeper mechanistic understanding, it would be interesting to expand the PCEnT studies to triplet systems. Triplets usually have longer lifetimes and are generally known to undergo Dexter energy transfer due to the weak oscillator strength of their transition that hinders Förster energy transfer. However, triplet experimental data on ESIPT compounds are scarce,^11,25–28^ especially since their triplet states are generally not emissive at room temperature. A recent paper in the literature reports triplet PCEnT, both intramolecular and intermolecular, with zinc porphyrins as energy donors and 3HF as energy acceptor.^29^ The assignment to PCEnT is based on indirect evidence only, and all the experiments are performed in polar protic solvents where the hydrogen bond of 3HF is weakened and, according to our results, PCEnT is disfavored. Nonetheless, this report is interesting, and additional studies could confirm whether the observed mechanism is indeed triplet PCEnT.

In conclusion, the observation of PCEnT in a bimolecular system expands our knowledge of this type of reaction, but further work is needed to establish a detailed understanding of it and its potential importance and applications.

## Supplementary Material



## Notes

Materials and methods, additional fluorescence and flash photolysis experiments (PDF)

## References

[1] Mirkovic T., Ostroumov E. E., Anna J. M., Van Grondelle R., Govindjee, Scholes G. D. (2017). Light Absorption
and Energy Transfer in the Antenna Complexes of Photosynthetic Organisms. Chem. Rev..

[2] Scholes G. D., Fleming G. R., Olaya-Castro A., Van Grondelle R. (2011). Lessons from
Nature about Solar Light Harvesting. Nat. Chem..

[3] Rimgard B.
P., Tao Z., Parada G. A., Cotter L. F., Hammes-Schiffer S., Mayer J. M., Hammarström L. (2022). Proton-Coupled Energy Transfer in
Molecular Triads. Science.

[4] Tomin V. I., Demchenko A. P., Chou P. T. (2015). Thermodynamic vs. Kinetic Control
of Excited-State Proton Transfer Reactions. J. Photochem. Photobiol. C: Photochemistry Reviews.

[5] Hammes-Schiffer S. (2015). Proton-Coupled
Electron Transfer: Moving Together and Charging Forward. J. Am. Chem. Soc..

[6] Tyburski R., Liu T., Glover S. D., Hammarström L. (2021). Proton-Coupled Electron Transfer
Guidelines, Fair and Square. J. Am. Chem. Soc..

[7] Cui K., Hammes-Schiffer S. (2024). Theory for
Proton-Coupled Energy Transfer. J. Chem. Phys..

[8] Parada G. A., Markle T. F., Glover S. D., Hammarström L., Ott S., Zietz B. (2015). Control over Excited
State Intramolecular Proton Transfer
and Photoinduced Tautomerization: Influence of the Hydrogen-Bond Geometry. Chem.—Eur. J..

[9] Mcmorrow D., Kasha M. (1984). Intramolecular Excited-State
Proton Transfer in 3-Hydroxyflavone.
Hydrogen-Bonding Solvent Perturbations. J. Phys.
Chem..

[10] Martinez M. L., Cooper W. C., Chou P.-T. (1992). A Novel Excited-State Intramolecular
Proton Transfer Molecule, 10-Hydroxybenzo [h] Quinoline. Chem. Phys. Lett..

[11] Dick B. (1990). AM1 and INDO/S
Calculations on Electronic Singlet and Triplet States Involved In
Excited-State Intramolecular Proton Transfer of 3-Hydroxyflavone. J. Phys. Chem..

[12] Bellucci M. A., Coker D. F. (2012). Molecular Dynamics of Excited State Intramolecular
Proton Transfer: 3-Hydroxyflavone in Solution. J. Chem. Phys..

[13] Chang X. P., Fan F. R., Zhao G., Ma X., Zhang T. S., Xie B. (2023). Bin. CASPT2//CASSCF Studies on Mechanistic
Photophysics of 3-Hydroxyflavone. Chem. Phys..

[14] Zhou P., Han K. (2018). Unraveling the Detailed
Mechanism of Excited-State Proton Transfer. Acc. Chem. Res..

[15] Protti S., Mezzetti A. (2015). Solvent Effects on
the Photophysics and Photoreactivity
of 3-Hydroxyflavone: A Combined Spectroscopic and Kinetic Study. J. Mol. Liq..

[16] Ida K., Sakai H., Ohkubo K., Araki Y., Wada T., Sakanoue T., Takenobu T., Fukuzumi S., Hasobe T. (2014). Electron-Transfer
Reduction Properties and Excited-State Dynamics of Benzo­[Ghi]­Peryleneimide
and Coroneneimide Derivatives. J. Phys. Chem.
C.

[17] Woolfe G. J., Thistlethwaite P. J. (1981). Direct Observation of Excited State Intramolecular
Proton Transfer Kinetics in 3-Hydroxyflavone. J. Am. Chem. Soc..

[18] Slifkin M. A., Walmsley R. H. (1971). Triplet States of
Polycyclic Aromatic Hydrocarbons
in Fluid Solution and in the Solid State. Photochem.
Photobiol..

[19] Cotter L. F., Parada G. A., Bhide R., Rimgard B. P., Mayer J. M., Hammarström L. (2025). Evidence for
Competing Proton-Coupled Reaction Pathways
of Molecular Triads in a Low-Polarity Solvent. J. Phys. Chem. A.

[20] Kinka G. W., Faulkner L. R. (1976). Wurster’s
Blue as a Fluorescence Quencher for
Anthracene, Perylene, and Fluoranthene. J. Am.
Chem. Soc..

[21] Das S., Chakrabarty S., Chattopadhyay N. (2020). Origin of Unusually High Fluorescence
Anisotropy of 3-Hydroxyflavone in Water: Formation of Probe-Solvent
Cage-like Cluster. J. Phys. Chem. B.

[22] Chou P.-T., Wei C.-Y. (1996). Photophysics of 10-Hydroxybenzo­[h]­Quinoline in Aqueous
Solution. J. Phys. Chem..

[23] Böhnke H., Bahrenburg J., Ma X., Röttger K., Näther C., Rode M. F., Sobolewski A. L., Temps F. (2018). Ultrafast Dynamics
of the ESIPT Photoswitch *N*-(3-Pyridinyl)-2-Pyridinecarboxamide. Phys. Chem. Chem. Phys..

[24] Spörkel L., Jankowska J., Thiel W. (2015). Photoswitching of Salicylidene Methylamine:
A Theoretical Photodynamics Study. J. Phys.
Chem. B.

[25] Tokumura K., Kurauchi M., Yagata N., Itoh M. (1996). Phototautomerization
of 3-Hydroxyflavone in the Lowest Triplet State. Chem. Phys. Lett..

[26] Martinez M. L., Studer S. L., Chou P. T. (1990). Direct Evidence
of the Triplet-State
Origin of the Slow Reverse Proton Transfer Reaction of 3-Hydroxyflavone. J. Am. Chem. Soc..

[27] Zhao X., Chen M. (2011). A TDDFT Study on the
Singlet and Triplet Excited-State Hydrogen Bonding
and Proton Transfer of 10-Hydroxybenzo­[h]­Quinoline (HBQ) and 7,9-Diiodo-10-
Hydroxybenzo­[h]­Quinoline (DIHBQ). Chem. Phys.
Lett..

[28] Iijima T., Momotake A., Shinohara Y., Sato T., Nishimura Y., Arai T. (2010). Excited-State Intramolecular
Proton Transfer of Naphthalene-Fused
2-(2’-Hydroxyaryl)­Benzazole Family. J.
Phys. Chem. A.

[29] Ramundo A., Janoš J., Muchová L., Šranková M., Dostál J., Kloz M., Vítek L., Slavíček P., Klán P. (2024). Visible-Light-Activated
Carbon Monoxide Release from Porphyrin-Flavonol Hybrids. J. Am. Chem. Soc..

