# Supplementary material for: Direct Evidence of Bimolecular Proton-Coupled Energy Transfer at Room Temperature
Source: J Am Chem Soc. 2025 Jul 3;147(28):24181–5. doi: 10.1021/jacs.5c05126 (PMC12272677; doi:10.1021/jacs.5c05126)
Supplement: Supplementary file 1 [file ja5c05126_si_001.pdf]

Supplementary information to:
Direct evidence of bimolecular Proton-Coupled Energy Transfer at room temperature

Andrea Rosichini, Giorgio Scattolini, Leif Hammarström*

Department of Chemistry, Ångström Laboratory, Uppsala University, Box 523, 75120 Uppsala, Sweden.

Index

Materials and methods 2
- Materials..... 2
- Absorption and fluorescence spectroscopy 2
- Laser flash photolysis 2

Characterization of donor-acceptor systems in CTAC micelles..... 3

Fluorescence quenching experiments..... 4
- Perylene + P5Q 4
- Perylene + P6Q 4
- Perylene + HBQ..... 5
- Perylene + 3HF..... 5
- Benzo[ghi]perylene + 3HF/P5Q/P6Q/HBQ..... 5
- Benzo[ghi]perylene + P5Q..... 7
- Benzo[ghi]perylene + 3HF 8
- Benzo[ghi]perylene + MeO3HF 9
- Benzo[ghi]perylene + 3HF in CTAC micelles..... 9
- Benzo[ghi]perylene + 3HF in ethanol 9

Laser flash photolysis experiments 10

TA features of reduced benzo[ghi]perylene 14

Benzo[ghi]perylene + 3HF quenching rate constants calculation..... 14

Polarity dependence of 3HF emission..... 14

References..... 15

Materials and methods

Materials

Benzo[ghi]perylene (Sigma Aldrich), Perylene (Sigma Aldrich), p-anisidine (Sigma Aldrich), 3HF (Tokyo Chemical Industry) and HBQ (Tokyo Chemical Industry) were purchased from the respective companies and used without further purification.
P5Q and P6Q were synthesised by Parada et al. for a previous project, and the synthesis is described elsewhere[1].
For experiments with benzo[ghi]perylene, donor and acceptor were dissolved directly in the solvent and sonicated for 10 minutes if necessary. THF, toluene and decane were purchased from Sigma Aldrich. Acetonitrile and ethanol (for spectroscopy) was purchased from Merck.
For the experiments with perylene in micelles, samples were prepared by dissolving the donor and the acceptor in THF for spectroscopy (Merck) and by then diluting those solutions in a 7.6 mM aqueous solution of cetyltrimethylammonium chloride (CTAC). The final occupation degree of perylene in the micelles was kept in the range 0.2 to 0.4 (calculated from perylene absorbance). The occupation degree of the acceptors is reported for each individual experiment. The CTAC solution was previously prepared by diluting 1:100 a 25% w/w CTAC/water stock solution (Sigma Aldrich).

Absorption and fluorescence spectroscopy

Steady-state absorption measurements were performed with a Cary 5000 spectrophotometer (Varian) and Cary 50 spectrophotometer (Varian).
Steady-state emission measurements were performed in right-angle mode with a Jobin Yvon Fluorolog fluorimeter (Horiba).
All measurements were performed in a 1 cm quartz cuvette.
In the experiments with benzo[ghi]perylene, the solutions were purged with argon for 10 minutes before measuring to avoid quenching from oxygen.

Laser flash photolysis

Laser flash photolysis was measured using an EKSPLA Model NT342B as excitation source. 390 nm laser pulses with a power of 3 mJ/pulse and a nominal temporal width of 6ns, generated from the 355 nm third harmonic of the laser using a sum frequency generation crystal, were used to excite of the sample. A pulsed 450 W ozone-free Xe arc lamp was used as probe. The pump and the probe were positioned in a right-angle set up.
The spectra were acquired with the LP920 detection system (Edinburgh Instruments) using a symmetrical Czerny-Turner monochromator (TMS300) with 5 nm bandwidth. Detectors were selected for either single kinetic traces (LP900 photomultiplier, with Tektronix TDS3012C oscilloscope) or full spectra (Andor SH720 ICCD camera). The gate width for the TA spectra was kept at 10 ns in all measurements. The final spectra and traces were produced with the appropriate software (L900). All the TA spectra are presented without subtraction of spontaneous emission induced by the laser pulse. All measurements were averaged over four shots. The solutions were purged with argon for 10 minutes before measuring to avoid quenching from oxygen.

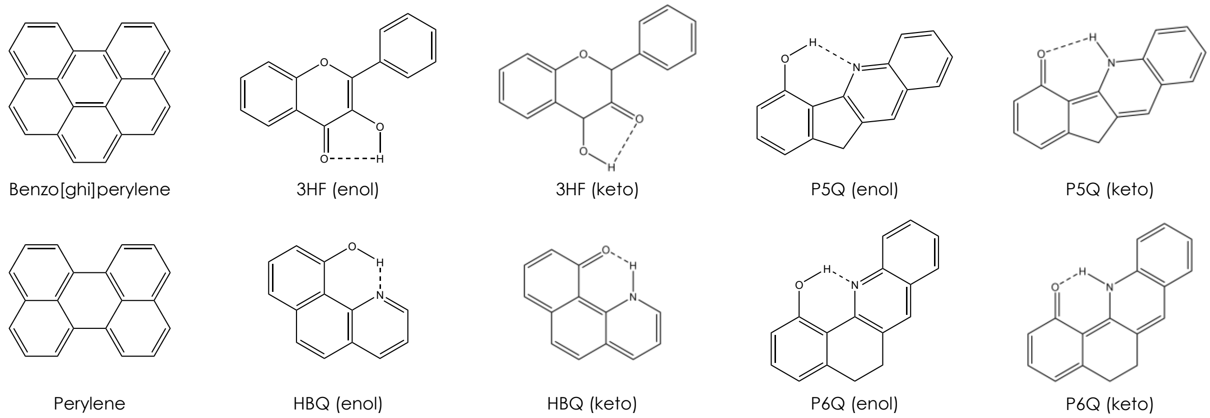

Figure S1: Molecular structures of energy donors and acceptors. Both enol and keto forms of the acceptors are shown.

Characterization of donor-acceptor systems in CTAC micelles

We used the changes in absorption spectra to assess the successful inclusion of the acceptors inside the CTAC micelles.

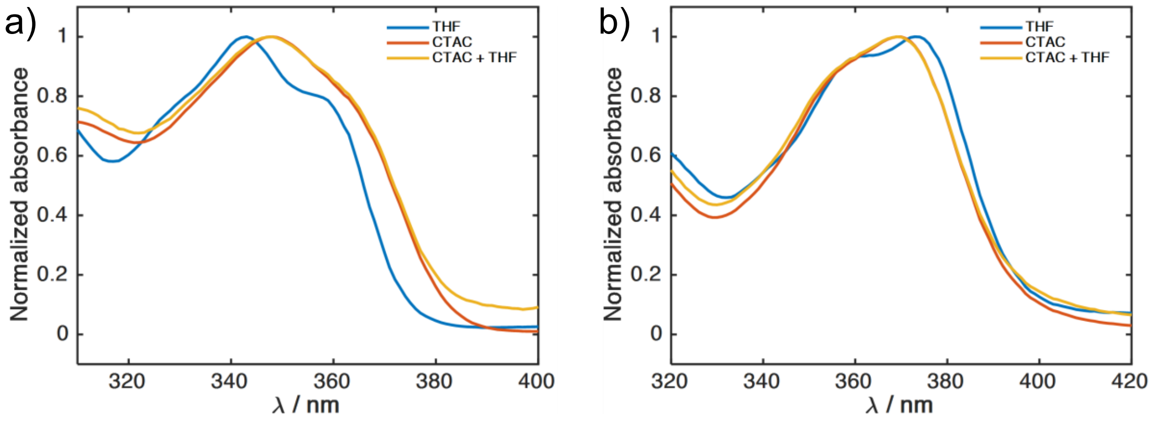

Figure S2: Absorption spectra of a) 3HF and b) HBQ in THF, CTAC, and CTAC with 1% THF.

Fluorescence quenching experiments

Perylene + P5Q

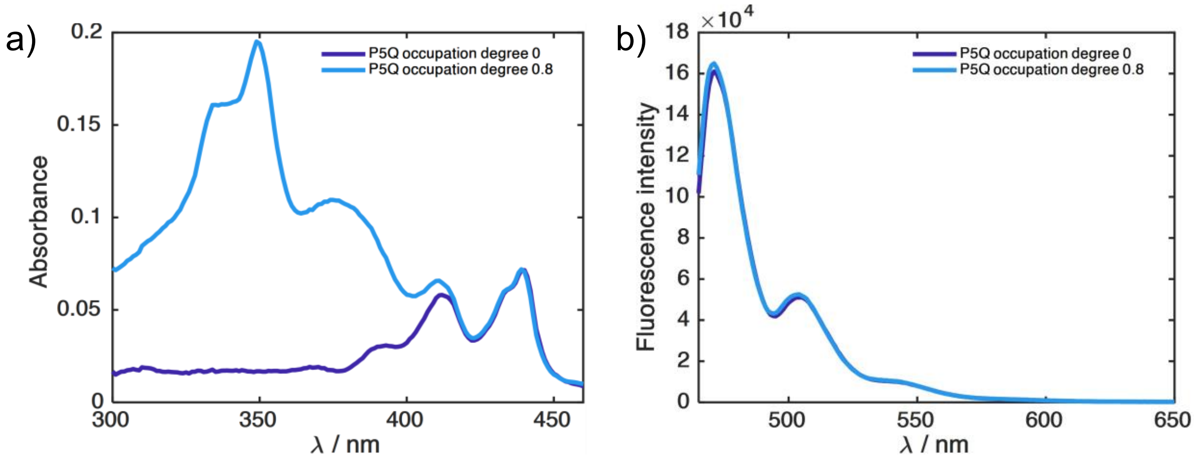

Figure S3: a) Absorption and b) emission spectra of perylene in CTAC micelles in absence and in presence of P5Q as a quencher. Samples were excited at 440 nm. No quenching is observed.

Perylene + P6Q

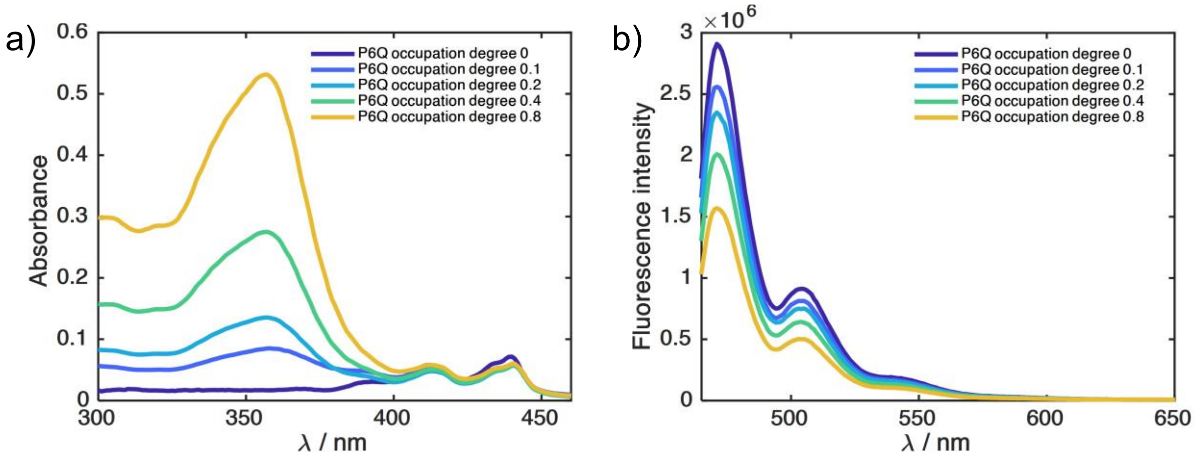

Figure S4: a) Absorption and b) emission spectra of perylene in CTAC micelles in absence and in presence of different concentrations of P6Q as a quencher. Samples were excited at 440 nm. Perylene emission is quenched, but no enhanced emission from P6Q is observed.

Perylene + HBQ

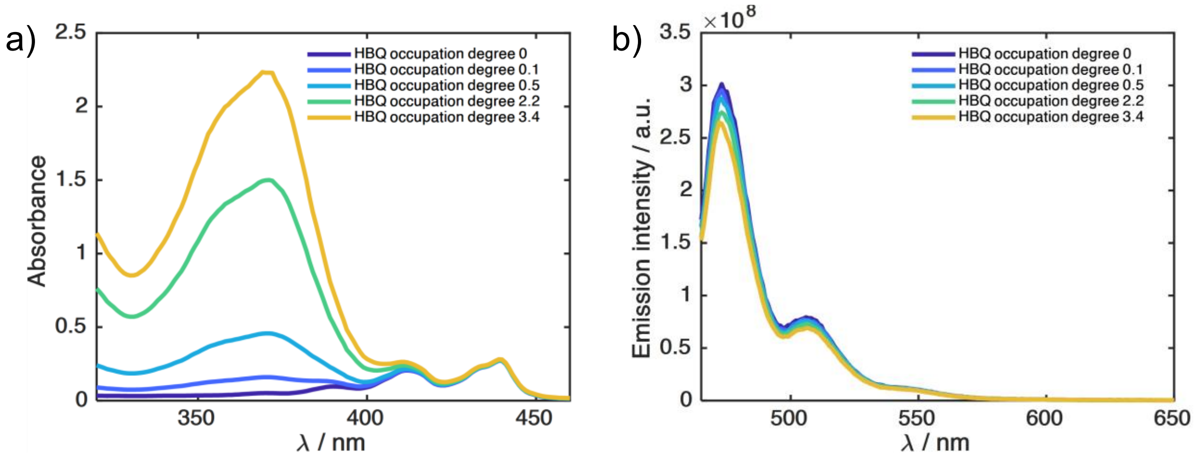

Figure S5: a) Absorption and b) emission spectra of perylene in CTAC micelles in absence and in presence of different concentrations of HBQ as a quencher. Samples were excited at 440 nm. Perylene emission is quenched at high concentrations of HBQ, but no enhanced emission from HBQ is observed

Perylene + 3HF

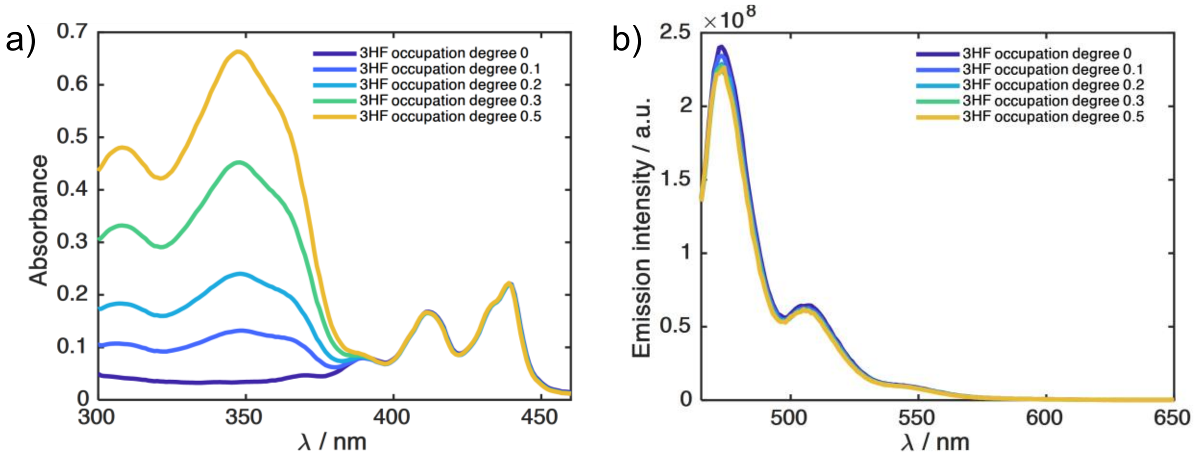

Figure S6: a) Absorption and b) emission spectra of perylene in CTAC micelles in absence and in presence of different concentrations of 3HF as a quencher. Samples were excited at 440 nm. Perylene emission is minimally quenched, but no enhanced emission from 3HF is observed.

Benzo[ghi]perylene + 3HF/P5Q/P6Q/HBQ

P6Q and HBQ could not be measured with benzo[ghi]perylene since their absorption tail at high concentration (500 µM) overlaps and covers completely benzo[ghi]perylene absorption.

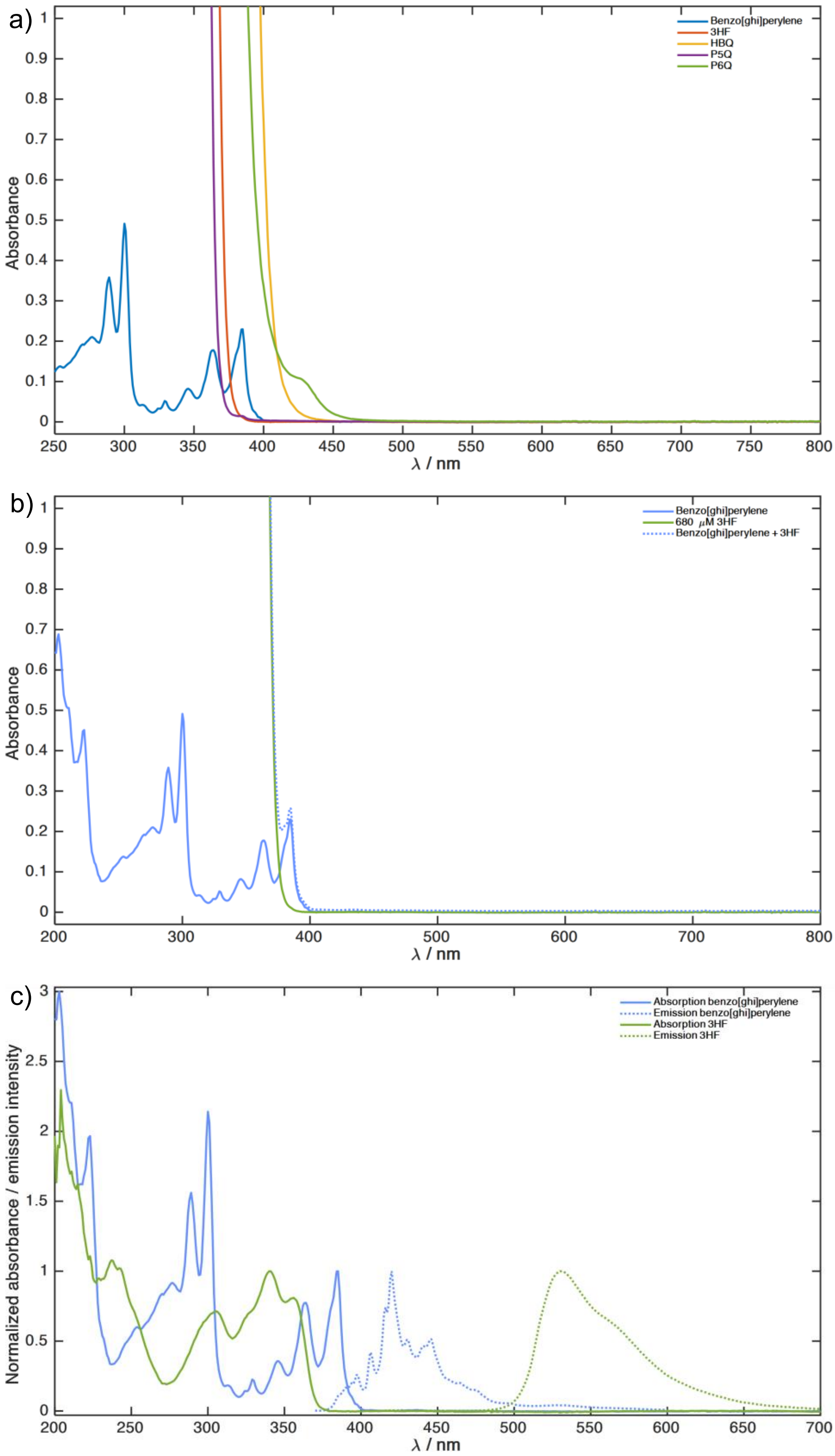

Figure S7: a) Absorption spectra of benzo[ghi]perylene, 3HF, P5Q, HBQ and P6Q in decane. b) Absorption spectra in decane of benzo[ghi]perylene, 680 µM 3HF and of benzo[ghi]perylene in the presence of 680 µM 3HF. No additional feature is observed in the spectrum with both species indicating no detectable formation of excimers or aggregates of the two molecules. c) Normalized absorption and emission spectra of benzo[ghi]perylene and 3HF in decane. For the emission spectra, benzo[ghi]perylene was excited at 350 nm, while 3HF was excited at 378 nm.

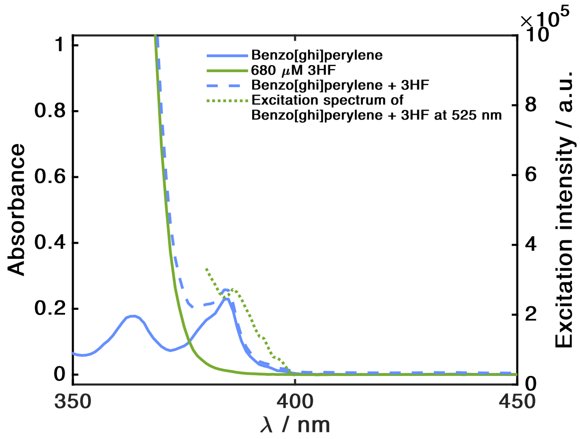

Figure S8: Absorption spectra of in decane of benzo[ghi]perylene, 680 µM 3HF and of benzo[ghi]perylene in the presence of 680 µM 3HF overlapped with the excitation spectrum measured at 525 nm in front face mode in decane of the binary mixture. It is possible to observe that the peak at 390 nm of benzo[ghi]perylene is present in the excitation spectrum as well, indicating that energy transfer is occurring between the two species.

Benzo[ghi]perylene + P5Q

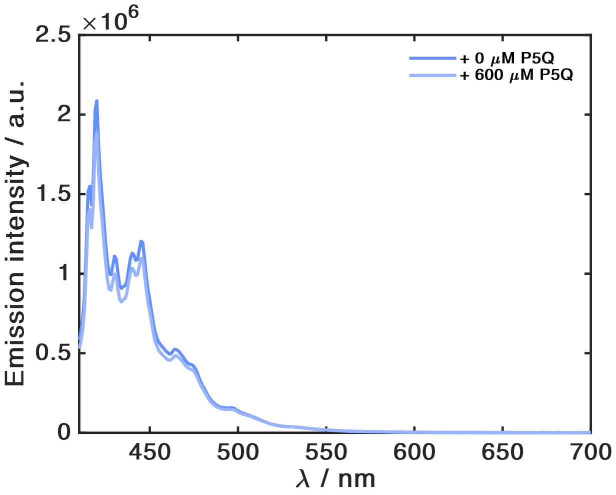

Figure S9: Emission spectra of benzo[ghi]perylene in decane in absence and in presence of 600 µM of P5Q as a quencher. Perylene emission is minimally quenched, but no enhanced emission from P5Q is observed. Samples were excited at 390 nm.

Benzo[ghi]perylene + 3HF

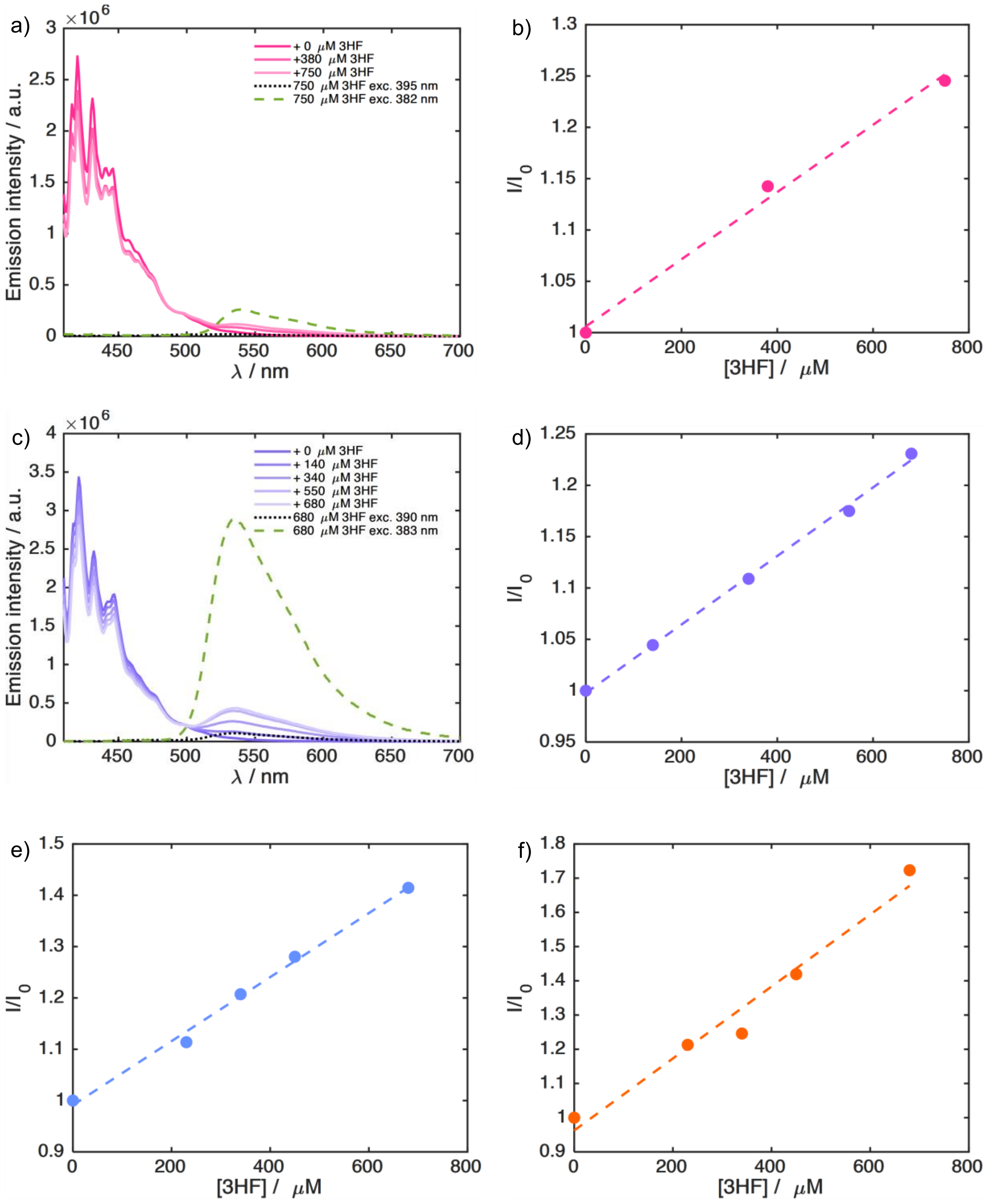

Figure S10: a) Emission spectra of benzo[ghi]perylene in THF in absence and in presence of different concentrations of 3HF as a quencher. Dotted and dashed lines in panel a) show the emission of 3HF on its own at the excitation wavelength used for the donor (390 nm) and at an excitation wavelegth where the acceptor have the same absorbance as the donor at 390 nm, respectively. b) Stern-Volmer plot obtained from data shown in panel a). c) Emission spectra of benzo[ghi]perylene in toluene in absence and in presence of different concentrations of 3HF as a quencher. Dotted and dashed lines in panel a) show the emission of 3HF on its own at the excitation wavelength used for the donor (390 nm) and at an excitation wavelegth where the acceptor have the same absorbance as the donor at 390 nm, respectively. d) Stern-Volmer plot obtained from data shown in panel c). e) Stern-Volmer plot obtained for the data in figure 2a. f) Stern-Volmer plot obtained for the data in figure 2b.

Benzo[ghi]perylene + MeO3HF

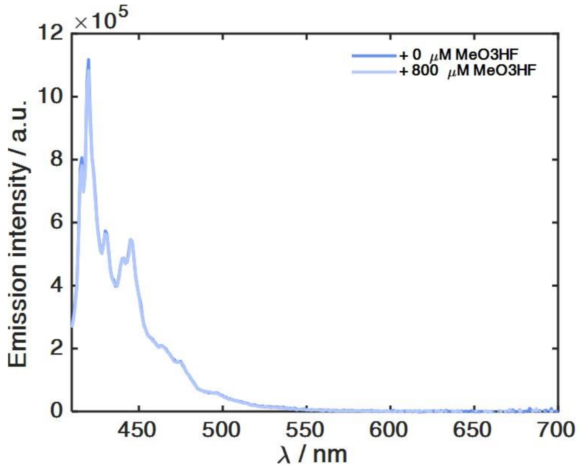

Figure S11: Emission spectra of benzo[ghi]perylene in decane in absence and in presence of MeO3HF as a quencher. Samples were excited at 390 nm.No quenching is observed.

Benzo[ghi]perylene + 3HF in CTAC micelles

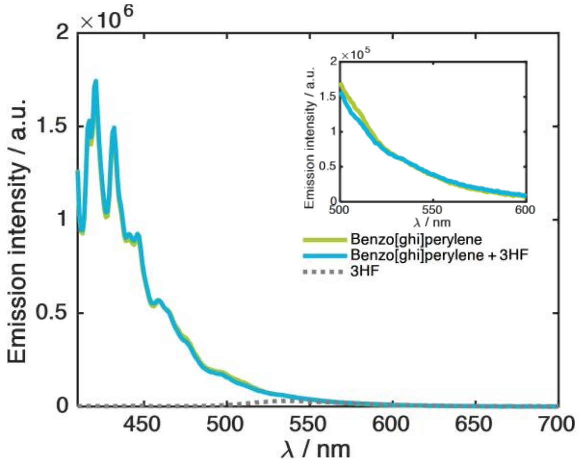

Figure S12: Emission spectra of benzo[ghi]perylene in CTAC micelles in absence and in presence of 3HF as a quencher. Samples were excited at 390 nm. No quenching is observed.

Benzo[ghi]perylene + 3HF in ethanol

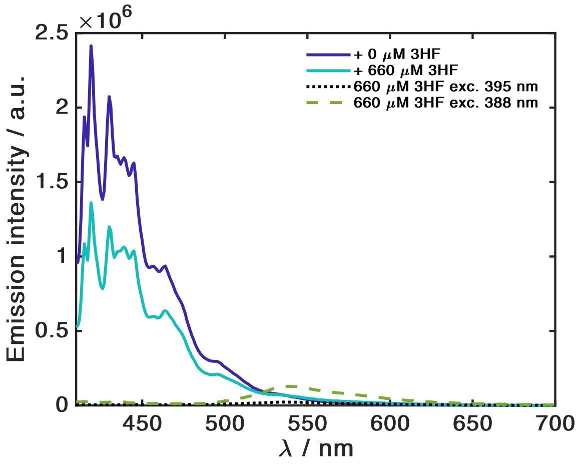

Figure S13: Emission spectra of benzo[ghi]perylene in ethanol in absence and in presence of 3HF. . Samples were excited at 390 nm. The fluorescence intensity is quenched, but no enhanced emission is observed.

Laser flash photolysis experiments

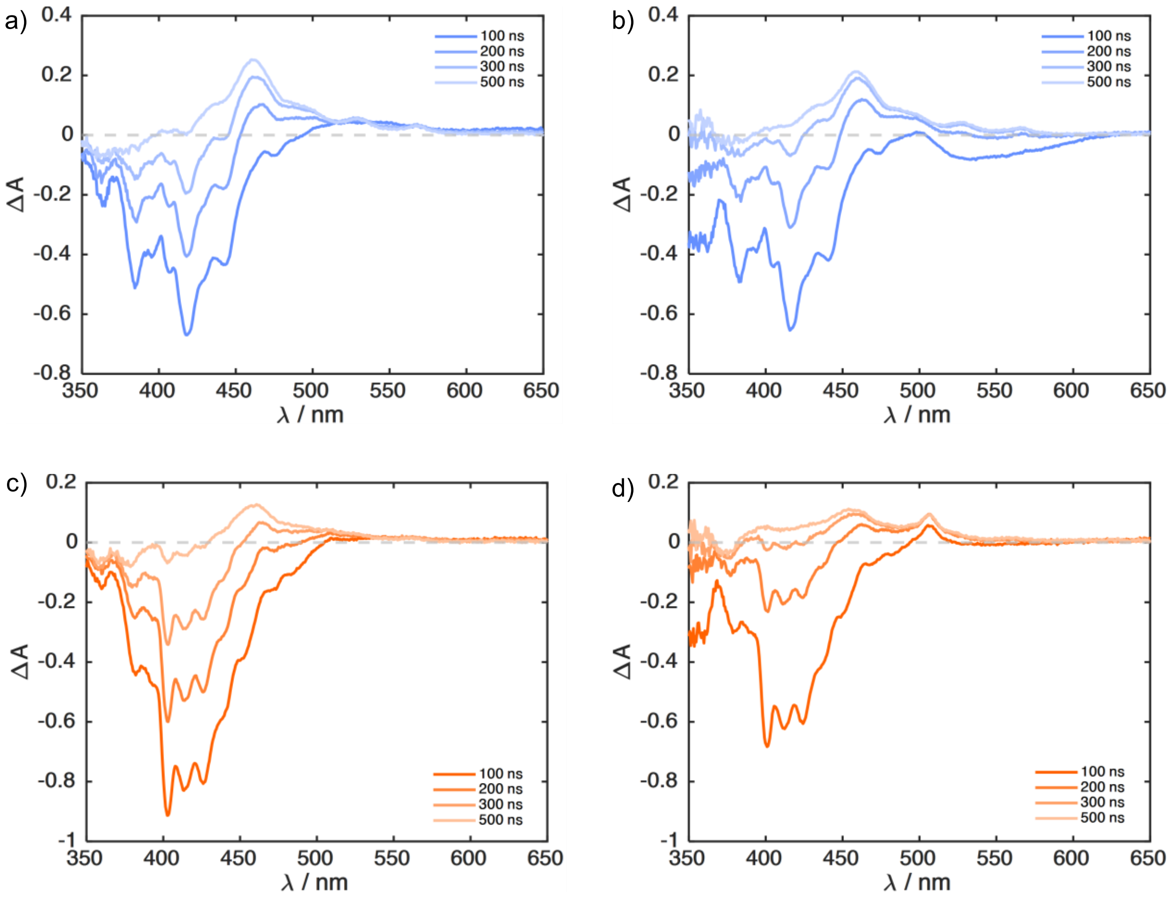

Figure S14: Panels a) and b): Transient absorption spectra of benzo[ghi]perylene in decane (excitation at 390 nm) in absence a) and in presence b) of 680 µm 3HF as a quencher, measured on short timescales (100 ns to 500 ns). Panels c) and d): Transient absorption spectra of benzo[ghi]perylene in acetonitrile (excitation at 390 nm) in absence c) and in presence d) of 680 µm 3HF as a quencher, measured on short timescales (100 ns to 500 ns).

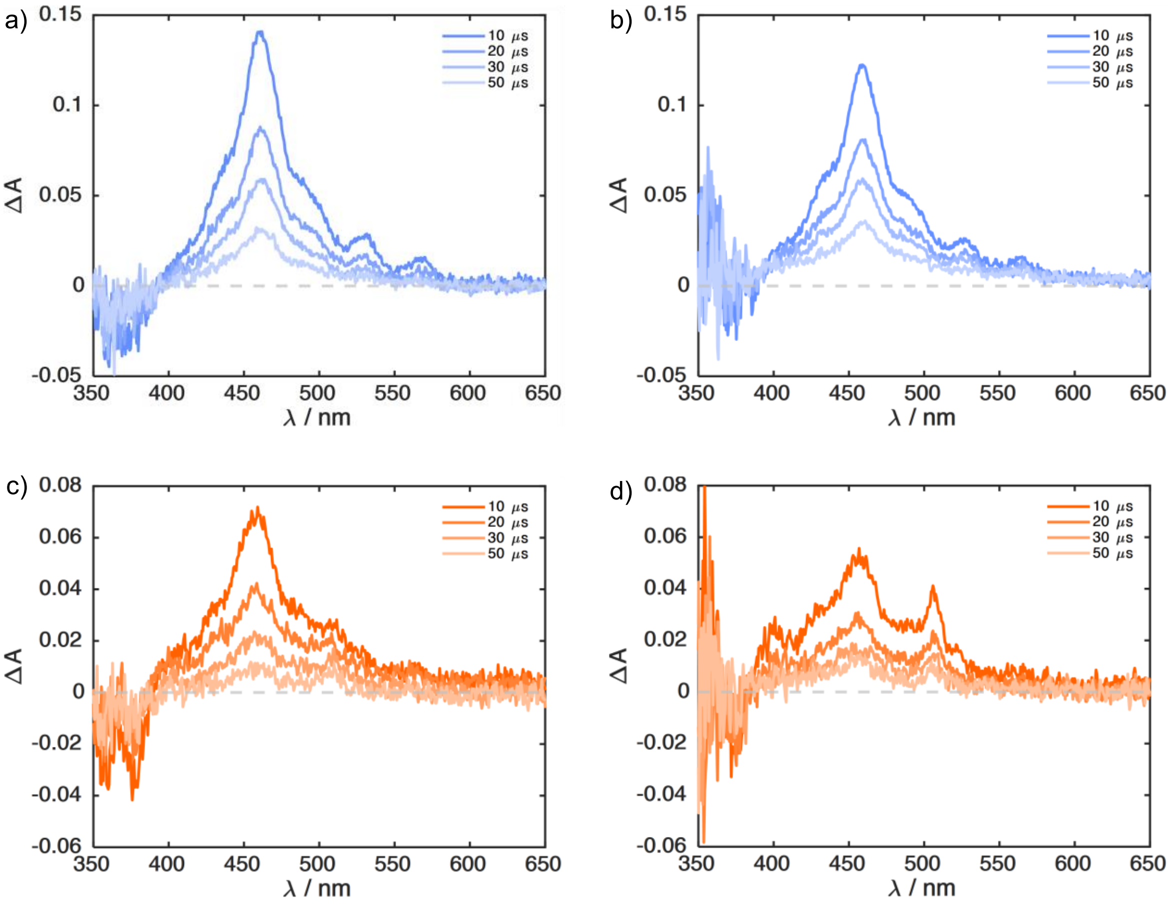

Figure S15: Panels a) and b): Transient absorption spectra of benzo[ghi]perylene in decane (excitation at 390 nm) in absence a) and in presence b) of 680 µm 3HF as a quencher, measured at long timescales (10 µs to 50 µs). Panels c) and d): Transient absorption spectra of benzo[ghi]perylene in acetonitrile (excitation at 390 nm) in absence c) and in presence d) of 680 µm 3HF as a quencher, measured on long timescales (10 µs to 50 µs).

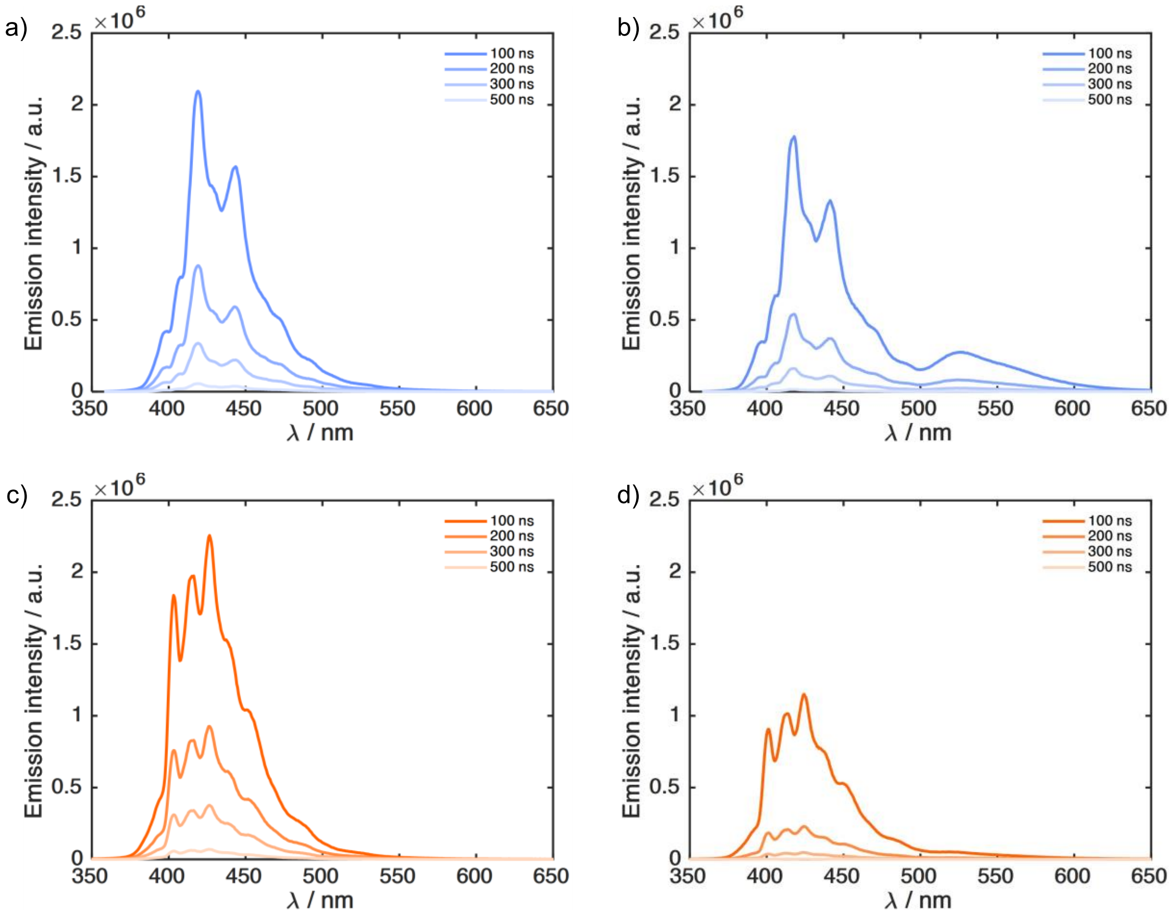

Figure S16: Panels a) and b): Transient emission spectra of benzo[ghi]perylene in decane (excitation at 390 nm) in absence a) and in presence b) of 680 µm 3HF as a quencher. Panels c) and d): Transient emission spectra of benzo[ghi]perylene in acetonitrile (excitation at 390 nm) in absence c) and in presence d) of 680 µm 3HF as a quencher.

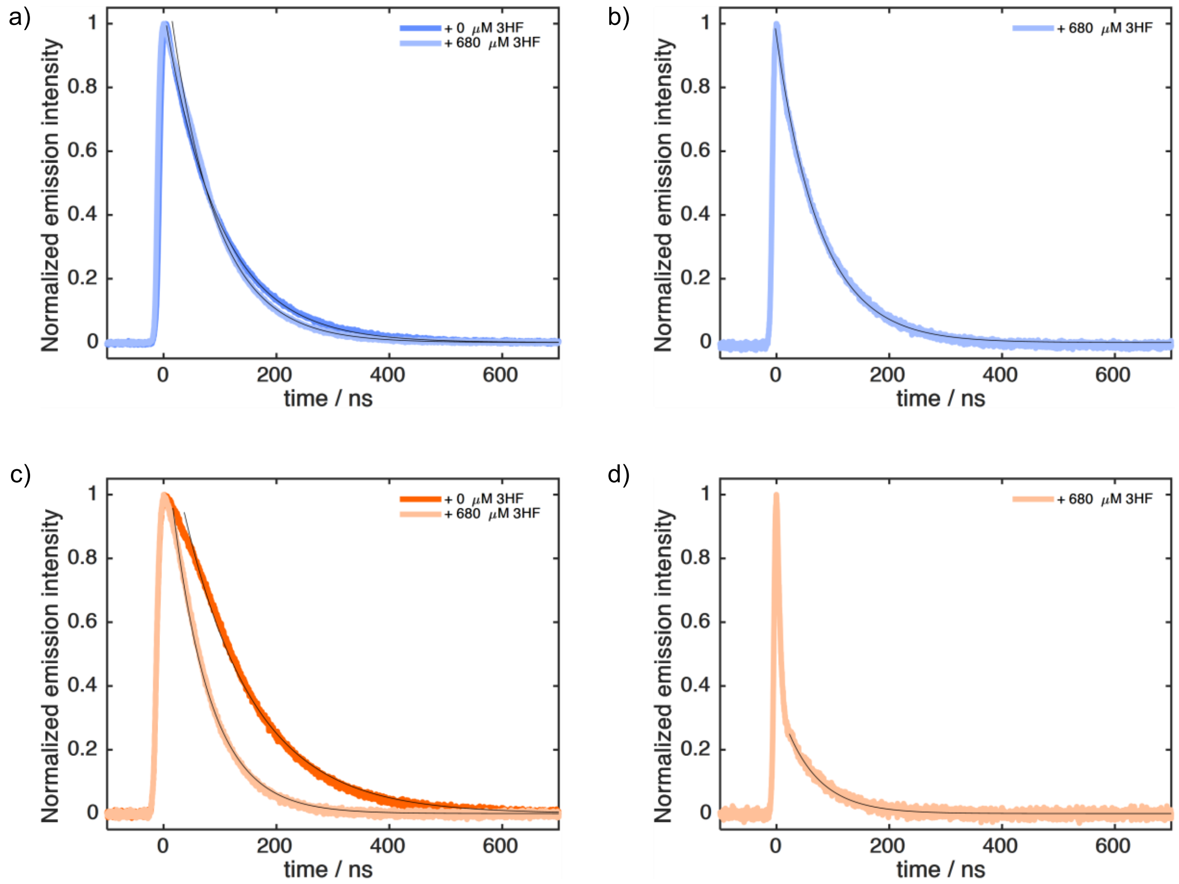

Figure S17: Panel a) Transient emission kinetics of benzo[ghi]perylene in decane (excitation at 390 nm) in absence and in presence of 680 µm 3HF as a quencher, measured at 450 nm (emission of benzo[ghi]perylene). Panel b) Transient emission kinetics of a solution of benzo[ghi]perylene and 680 µm 3HF as a quencher in decane. Kinetics were measured at 550 nm (emission of 3HF). Panel c): Transient emission kinetics of benzo[ghi]perylene in acetonitrile (excitation at 390 nm) in absence and in presence of 680 µm 3HF as a quencher, measured at 410 nm (emission of benzo[ghi]perylene). Panel d) Transient emission kinetics of a solution of benzo[ghi]perylene and 680 µm 3HF as a quencher in acetonitrile. Kinetics were measured at 525 nm (emission of 3HF).

TA features of reduced benzo[ghi]perylene

Benzo[ghi]perylene was reductively quenched in a flash photolysis experiment using 10 mM p-anisidine as a quencher. The TA spectra of the reduced form of benzo[ghi]perylene was recorded at different time delays from the laser pulse.

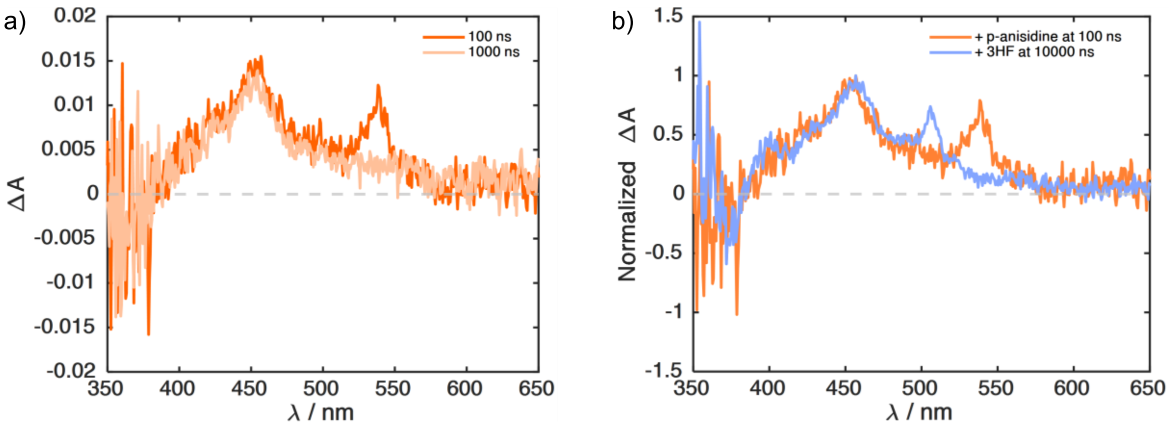

Figure S18: a) TA spectra of benzo[ghi]perylene (excitation at 390 nm) in the presence of p-anisidine at 100 and 1000 ns. b) Normalized TA spectra of benzo[ghi]perylene in acetonitrile in the presence of p-anisidine at 100 ns (orange) and in the presence of 3HF at 10000 ns (purple)

The peak between 500 nm and 550 nm corresponds to the reduced form of benzo[ghi]perylene. The ≈ 40 nm red shift between in the experiment with p-anisidine compared to the one with 3HF could be due to aggregation behaviour in the former conditions, caused by the 20 times higher concentration of quencher used.

Benzo[ghi]perylene + 3HF quenching rate constants calculation

Quenching rate constants from steady state were calculated from a linear fit of a Stern-Volmer plot using Stern Volmer equation:

$$\frac{I_0}{I} = k_q \tau_0 [3HF] + 1 = k_{SV} [3HF] + 1$$

where τ_0 is the unquenched lifetime obtained from transient emission. Note that τ_0 in Toluene and THF was not obtained, and τ_0 obtained in decane was used instead. Since τ_0 of benzo[ghi]perylene has been reported to increase with solvent polarity, k_q in THF could be slightly underestimated.

Quenching rate constants from transient emission were calculated knowing that:

$$k_{obs} = k_0 + k_q [3HF]$$

Where $k_0 = 1/\tau_0$

Polarity dependence of 3HF emission

The ESIPT emission of 3HF has been reported to be strongly dependent on the polarity and on the hydrogen bonding properties of the solvent[2–4]. To estimate the effect of such solvent dependence in our experiments, we measured absorption and emission spectra of 3HF in a range of solvents of different polarities, both aprotic and protic, including all the solvents used in our experiments. A concentrated 3HF sample was prepared in THF. 1 ml aliquotes were then prepared in different vials

and evaporated. Finally, 3HF was redissolved the same volume of of the different solvents to ensure that all the samples had the same concentration.

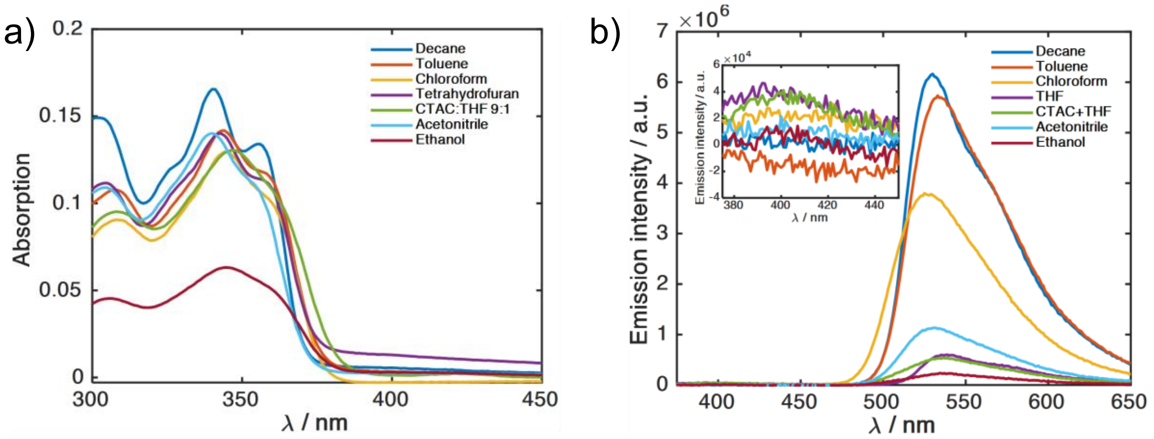

Figure S19: a) Absorption and b) emission spectra of 3HF in different solvents

References

(1) Parada, G. A.; Glover, S. D.; Orthaber, A.; Hammarström, L.; Ott, S. Hydrogen Bonded Phenol-Quinolines with Highly Controlled Proton-Transfer Coordinate. *European J Org Chem* **2016**, *2016* (20), 3365–3372.

(2) Mcmorrow, D.; Kasha, M. Intramolecular Excited-State Proton Transfer in 3-Hydroxyflavone. Hydrogen-Bonding Solvent Perturbations. *J. Phys. Chem* **1984**, *88*, 2235–2243.

(3) Protti, S.; Mezzetti, A. Solvent Effects on the Photophysics and Photoreactivity of 3-Hydroxyflavone: A Combined Spectroscopic and Kinetic Study. *J Mol Liq* **2015**, *205*, 110–114.

(4) Das, S.; Chakrabarty, S.; Chattopadhyay, N. Origin of Unusually High Fluorescence Anisotropy of 3-Hydroxyflavone in Water: Formation of Probe-Solvent Cage-like Cluster. *J. Phys. Chem. B* **2020**, *124* (1), 173–180.
